# Developmental toxicity of fluconazole and 1,2,4-triazole in *Xenopus laevis*

**DOI:** 10.1038/s41598-025-30992-5

**Published:** 2025-12-06

**Authors:** Barbora Riesova, Lorena Agostini Maia, Renata Hesova, Nikola Peskova, Petr Marsalek, Jana Blahova, Pavla Lakdawala, Jakub Harnos

**Affiliations:** 1 Department of Animal Protection and Welfare & Veterinary Public Health, University of Veterinary Sciences, Brno, Czech Republic; 2 Department of Experimental Biology, Masaryk University, Brno, Czech Republic; 3 Department of Infectious Diseases and Preventive Medicine, Veterinary Research Institute, Brno, Czech Republic

**Keywords:** Azole antifungals, Embryotoxicity, Morphometric analysis, Gene expression, Wnt/BMP signaling, Non-target aquatic vertebrates, Developmental biology, Molecular biology, Physiology, Zoology

## Abstract

Fluconazole (FLU) is a widely used antifungal agent frequently detected in surface waters because of its extensive use in medicine, agriculture, and personal care products.

Despite concerns about its persistence and developmental toxicity in aquatic species, its effects on amphibians remain poorly understood. This study aimed to assess the developmental and molecular effects of FLU and its structural core, 1,2,4-triazole (TRI), in amphibian embryos. *Xenopus laevis* embryos were exposed to FLU or TRI and evaluated for mortality, hatching rate, heart rate, body length, malformation incidence, and changes in gene expression. Even at low micromolar concentrations, both azoles altered the expression of Wnt- and BMP-associated genes, indicating disruption of these signaling pathways. At higher micromolar concentrations, these molecular changes were accompanied by early signs of developmental abnormalities, which intensified at the highest doses. Observed phenotypes included reduced head size, altered skin pigmentation, prolonged body length, changes in heart rate, and mild digestive tract malformations. These findings demonstrate that even the core structural motif TRI can disrupt key developmental signaling pathways in vertebrate embryos, underscoring the need for closer monitoring of azole compounds in aquatic environments. Given the fundamental role of these pathways in vertebrate development, the results raise concerns about potential risks from long-term or prenatal exposure to azoles, in both environmental and clinical contexts.

**Supplementary Information:**

The online version contains supplementary material available at 10.1038/s41598-025-30992-5.

## Introduction

Azole antifungal compounds such as fluconazole (FLU) are widely used across human and veterinary medicine, agriculture, and the personal care industry^1–3^. Their therapeutic efficacy against fungal infections has made them indispensable in clinical practice, while their antifungal properties are also utilized in shampoos, soaps, skin creams, and agricultural fungicides^3–5^. A common structural feature of many azoles, including FLU, is the 1,2,4-triazole (TRI) ring—a heterocyclic motif essential for antifungal activity that is also present in numerous other pharmaceuticals and agrochemicals^6^. However, the extensive use of these compounds has resulted in their frequent detection in wastewater effluents and surface waters worldwide^7–11^. Conventional wastewater treatment plants often fail to remove them completely, leading to persistent low-level contamination. Measured nanomolar concentrations of azole compounds such as FLU, climbazole, and tebuconazole have been reported in rivers across Europe, Africa, and Asia, raising concerns about their ecotoxicological impact^10,12–16^.

Although originally developed for therapeutic use in humans and animals, azoles are increasingly recognized for their unintended effects on (non-target) organisms. Both FLU and TRI are highly photostable and degrade only slowly under natural sunlight or UV exposure (Suppl. Table 1). Consequently, FLU can persist in aquatic environments for months, and its transformation products such as TRI may remain for years, reflecting the environmental persistence of many azole fungicides^17,18^. The environmental stability of azoles raises concerns about their potential toxicity in aquatic organisms. Studies in zebrafish (*Danio rerio*) have shown that they can disrupt early development, causing pericardial edema, altered heart rate, and embryonic malformations^7,19^. Yet, little is known about their developmental effects in amphibians and higher vertebrates. Amphibians are particularly vulnerable to waterborne contaminants due to their permeable skin and aquatic life stages. The African clawed frog, *Xenopus laevis*, is a long-established and highly sensitive model organism that has made fundamental contributions to developmental and cell biology, as well as to ecotoxicology^20,21^. Its external fertilization, rapid development, and highly conserved genetic pathways with other vertebrates, including humans, make it especially suitable for assessing the impact of environmental chemicals^21^. This species also serves as the basis of the standardized FETAX (Frog Embryo Teratogenesis Assay—*Xenopus*) test, commonly applied to assess developmental toxicity in amphibians^22,23^.

In the present study, we hypothesized that fluconazole (FLU) and its main degradation product 1,2,4-triazole (TRI) may interfere with early vertebrate development by affecting conserved signaling pathways regulating embryogenesis. To test this hypothesis, we investigated the developmental toxicity of FLU and its structural core TRI in *Xenopus laevis* embryos. TRI was tested because azole fungicides such as FLU degrade into it, and it is a common contaminant of surface waters. The selected concentrations were chosen based on comparisons with other studies on zebrafish, with the lowest concentration reflecting environmentally relevant levels^9,10^. We measured mortality, hatching success, heart rate, body length, malformation frequency, and expression of key genes involved in early embryogenesis. Both compounds induced morphological and physiological changes, and at the molecular level, altered the expression of *xbra*, *xolloid, β-catenin*, *chordin* and *noggin*, which is consistent with modulation of Wnt (Wingless/Integrated) and BMP (Bone Morphogenetic Protein) signaling pathways. These results suggest that even simple triazole structures can interfere with conserved developmental mechanisms and raise important questions about the environmental and health safety of azole antifungals.

## Results

### Azole antifungals alter tadpole morphology

**Fig. 1:**
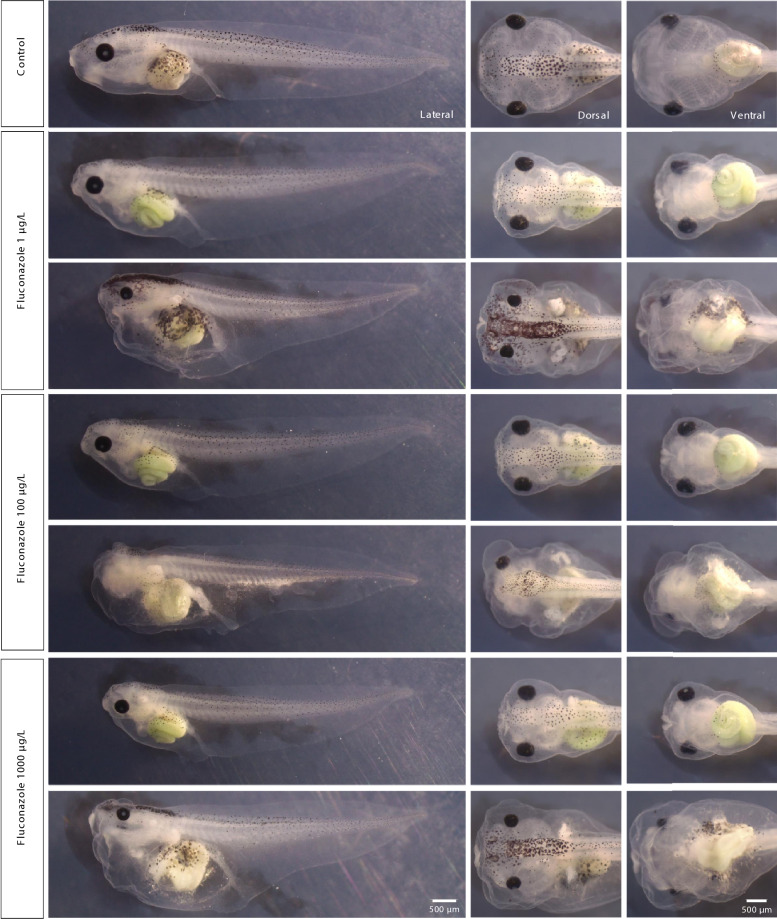
Representative images of *Xenopus laevis* embryos exposed to fluconazole (FLU). Embryos were treated from stage NF 3 to NF 45 with increasing concentrations of FLU (1, 100, and 1000 µg/L). Image acquisition and scoring followed FETAX criteria for morphological endpoints. Phenotypic abnormalities were observed at all tested concentrations, with most prominent features including reduced head size, eye deformities, craniofacial malformations, altered pigmentation, and gut abnormalities. Images were captured at NF stage 45 using a stereomicroscope. Scale bar: 500 µm.

**Fig. 2:**
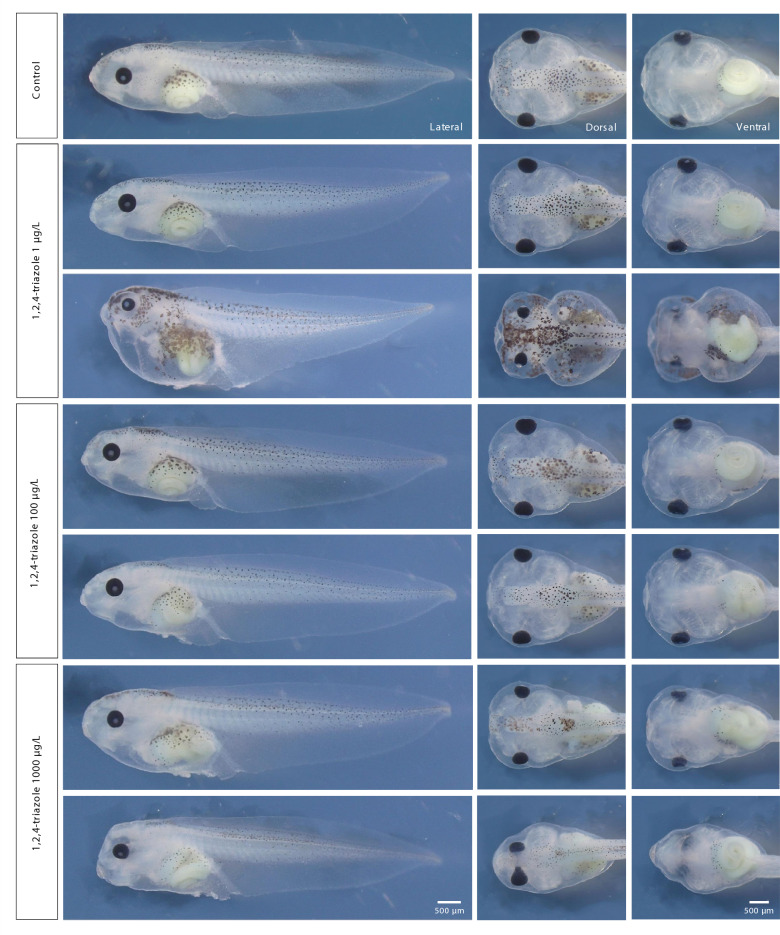
Representative images of *Xenopus laevis* embryos exposed to 1,2,4-triazole (TRI). Embryos were treated from stage NF 3 to NF 45 with 1, 100, and 1000 µg/L TRI. Phenotype scoring adhered to FETAX guidelines. A concentration-dependent increase in morphological abnormalities was observed, including smaller heads, eye spacing defects, changes in pigmentation, and intestinal malformations. Images were taken at stage NF 45. Scale bar: 500 µm.

To investigate the developmental toxicity of azole antifungals, we initially monitored the development of *Xenopus laevis* embryos during early stages but observed no overt morphological changes. Therefore, we focused our analysis on later time points, specifically at Nieuwkoop and Faber (NF) stage 45, when cumulative effects become more apparent. At this stage, we examined gross morphology following continuous exposure to 1–1000 µg/L of FLU and TRI. Representative phenotypes for both treatments are shown in Fig. 1 (FLU) and Fig. 2 (TRI).

Untreated control animals developed normally with well-formed heads, regular pigmentation, and a straight body axis, but exposed embryos exhibited noticeable morphological deviations. In FLU-treated groups, we observed several recurring abnormalities, including pigmentation defects—both hyperpigmentation and hypopigmentation—and craniofacial malformations such as reduced head size, edema and, in the most severe cases, misshapen anterior structures. Heart edema and visible gut malformations were also present in a subset of tadpoles (Fig. 1).

TRI exposure resulted in a similar but more variable range of phenotypes. Along with pigmentation defects, observed phenotypes included reduced eye spacing, even connected, and abnormal gut coiling intestines (Fig. 2).

Taken together, these observations suggest that both FLU and TRI interfere with normal tissue patterning and organogenesis in *Xenopus laevis* embryos.

### Dose-dependent differences in malformation incidence

**Fig. 3:**
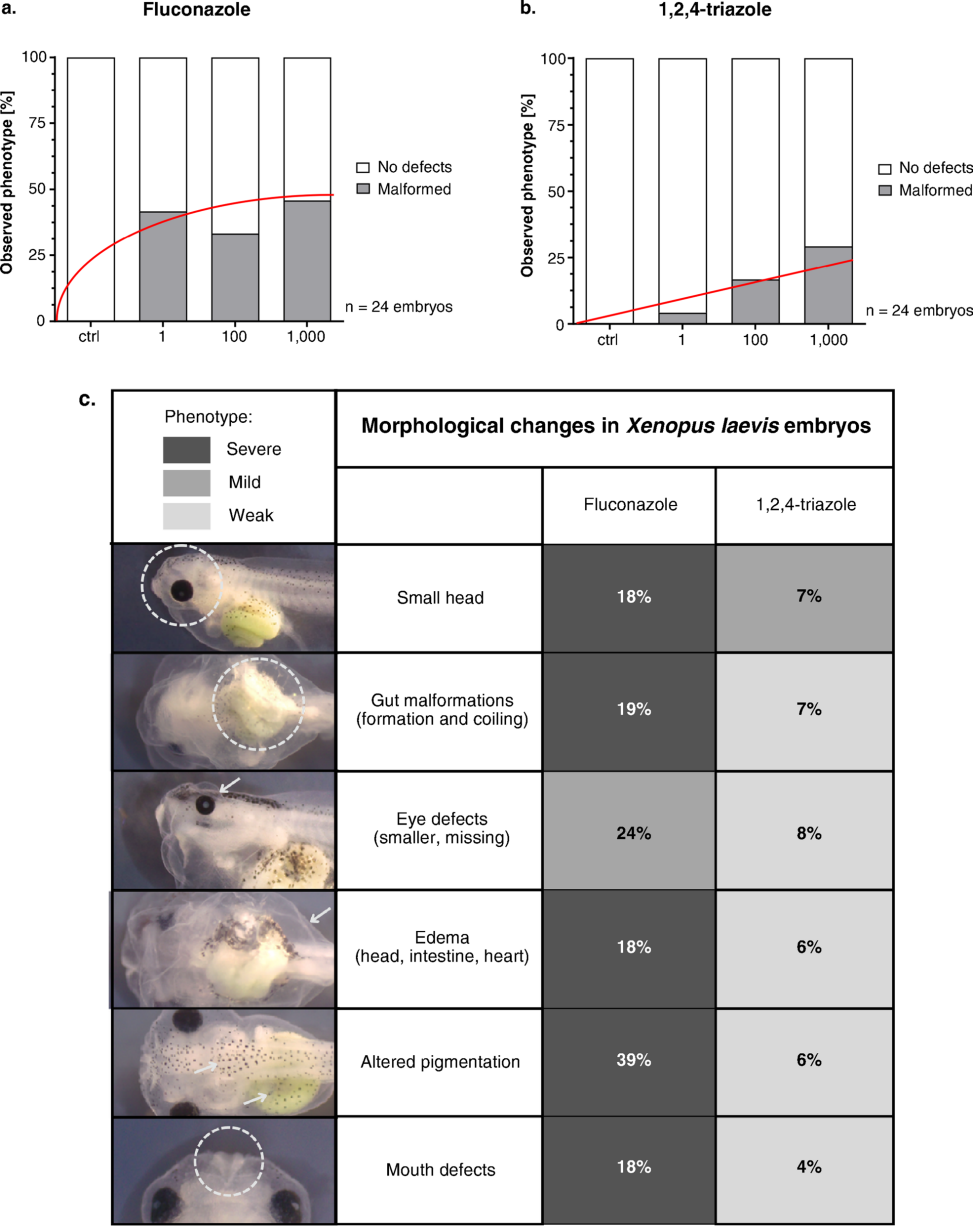
Detailed analysis of malformation incidence in embryos treated with FLU and TRI. (**A**–**B**) Bar plots show the percentage of embryos with developmental malformations at 120 hpf across increasing concentrations of FLU and TRI. While FLU caused a plateau in malformation incidence (~ 40%) starting from 1 µg/L, TRI exhibited a gradual, concentration-dependent increase in malformations, reaching ~ 25% at 1000 µg/L. Data represent 24 embryos per each experimental condition. Red lines represent type of dependency. (**C**) Incidence of malformations in *Xenopus laevis* embryos following exposure to FLU and TRI. The table presents the incidence of the main morphological alterations observed. Color intensity indicates a qualitative assessment of severity (dark gray: severe; gray: moderate; light gray: mild). Representative images of each phenotype are highlighted on the left. FLU exposure had a stronger effect on *Xenopus* development compared to TRI.

We next quantified the percentage of malformed tadpoles across increasing concentrations of both compounds to evaluate the dose–response relationship (Fig. 3A, B). FLU exposure resulted in a saturating malformation curve, with incidence plateauing at approximately 40% from the lowest tested concentration (1 μg/L) and remaining stable at higher doses. This suggests that even low concentrations of FLU are sufficient to trigger developmental disruptions and that the system reaches a response ceiling early.

In contrast, TRI exposure followed a clear dose-dependent trend. At 1 μg/L, only a small proportion of animals exhibited any malformation. As the concentration increased to 100 μg/L, the incidence rose to approximately 20%, and at 1000 μg/L, around 25% of the tadpoles displayed visible abnormalities. These findings indicate a progressive teratogenic effect of TRI, potentially linked to cumulative molecular disruptions at higher doses. The contrasting saturation-versus-linear trend observed for FLU and TRI, respectively, points to possible differences in their mechanisms of action or bioavailability in the embryo.

### Malformation types differ between fluconazole and triazole

Beyond quantifying malformation rates, we analyzed the spectrum of phenotypic defects to identify potential differences in the type and severity of abnormalities caused by each compound. The percentage of the main malformations observed, as well as, the phenotype severity analysis is reported in Fig. 3C and Suppl. Fig. 1.

FLU exposure most frequently resulted in altered pigmentation, craniofacial changes (reduced head size, head edema), cardiac edema, mouth defects, and intestinal abnormalities. Notably, darker upper flank pigmentation was observed specifically at 100 µg/L. These phenotypes were generally consistent across concentrations, suggesting that FLU triggers a characteristic malformation profile that appears even at low doses.

TRI exposure induced a broader but generally less frequent set of malformations. Craniofacial anomalies included reduced head size, reduced interocular distance, and asymmetry of the eyes or head. Intestinal defects ranged from mild to weak deformation. Pigmentation changes were diverse, encompassing dark patches, hypopigmentation, and star-like pigment patterns absent in controls or FLU-treated animals. Additional phenotypes unique to TRI included slight dorsal tail flexure and underdeveloped fins. These effects tended to become more prevalent at higher concentrations, with combined malformations occurring at 1000 µg/L.

These qualitative differences indicate that while both compounds disrupt overlapping developmental processes, FLU produces a narrower and more consistent malformation profile, whereas TRI has a more variable and concentration-dependent impact, potentially reflecting a broader disruption of axial patterning.

### Cardiac and morphometric effects suggest physiological disruption

**Fig. 4:**
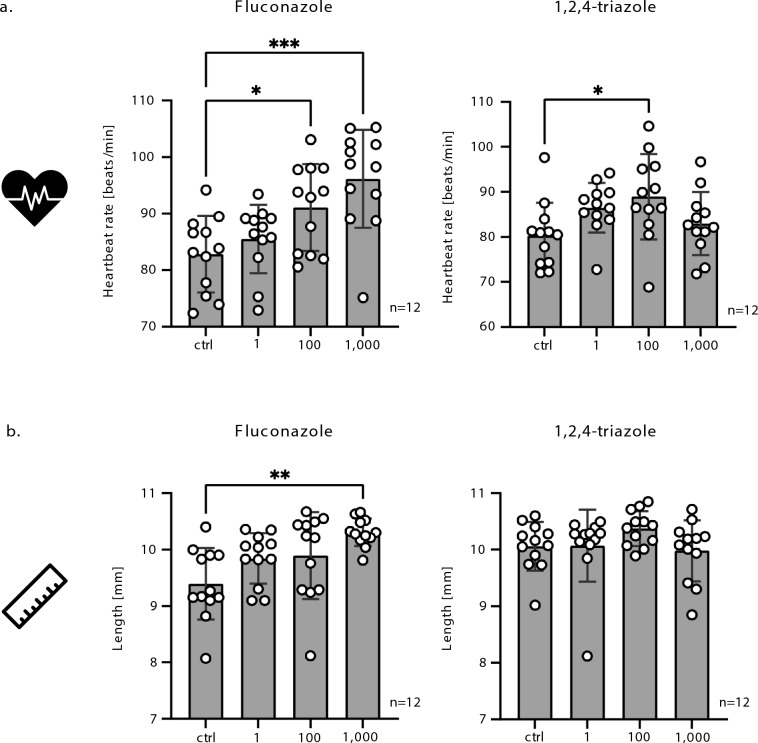
Morphometric and physiological endpoints in embryos exposed to FLU and TRI. (**A**) Body length measurements of embryos at 120 hpf showed that FLU significantly increased embryo length at higher doses, while TRI had no measurable effect. The values “1”, “10”, and “1000” below the graphs correspond to compound concentrations expressed in µg/L. (**B**) Heartbeat analysis revealed that both compounds increased heart rate at lower concentrations, but TRI led to a drop at 1000 µg/L. The values “1”, “10”, and “1000” below the graphs correspond to compound concentrations expressed in µg/L. Data represent mean ± SD from 12 individual embryos per group. See Material and Methods for more information about the measurement. Statistical significance was determined using ANOVA with Dunnett’s test. *, *p* < 0.05, **, *p* < 0.01, ***, *p* < 0.001.

To further explore the physiological consequences of azole exposure, we measured heart rate and body length in tadpoles at NF stage 45 (Fig. 4). Both compounds caused a significant increase in heart rate at most concentrations tested, indicating that cardiac development or autonomic regulation may be altered. Interestingly, at the highest TRI concentration (1000 μg/L), we observed a notable drop of heart rate, suggesting a possible toxic threshold beyond which physiological function becomes impaired.

Morphometric measurements revealed additional differences between the two compounds. FLU exposure led to a measurable elongation of the body axis, potentially reflecting disruptions in growth-regulating endocrine pathways and processes linked to mesoderm formation and muscle development, similar to patterns reported for cortisol and Bisphenol A exposure in fish and other model organisms^24,25^. In contrast, TRI-treated tadpoles did not show significant changes in body length, even at higher concentrations. These data point to distinct developmental effects of each compound—while both interfere with cardiac output, only FLU appears to affect axial elongation.

### Gene expression analysis reveals Wnt and BMP pathway disruption

**Fig. 5:**
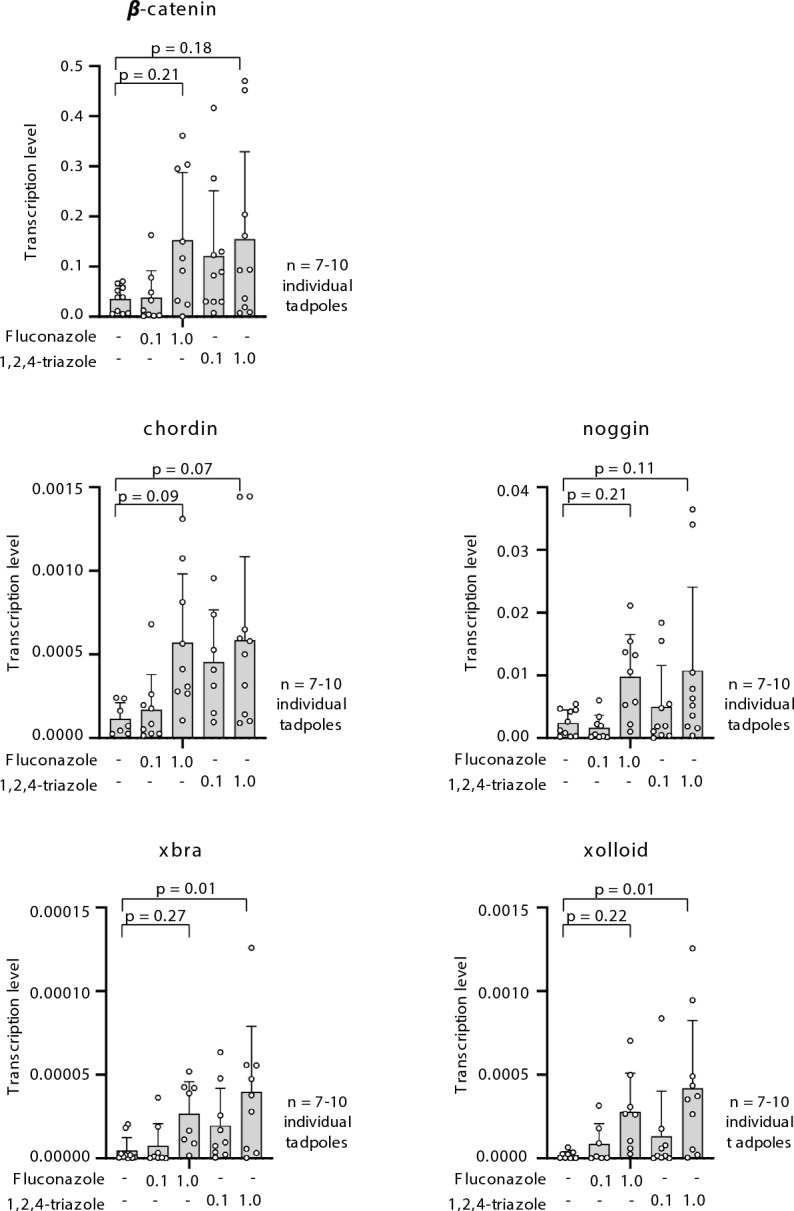
Gene expression analysis in *Xenopus laevis* embryos exposed to FLU and TRI. qPCR analysis of selected developmental genes (*β-catenin, noggin, chordin, xbra,* and *xolloid*) was performed at 120 hpf. The values “0.1” and “1.0” in the graph correspond to exposure concentrations of 0.1 µg/L and 1.0 µg/L, respectively. Significant upregulation was detected in embryos treated with both compounds, particularly TRI. These changes may reflect activation of Wnt signaling and modulation of BMP signaling. Data represent mean fold-change + SD from 7–10 embryos (ANOVA, Dunnett’s test).

To identify the molecular pathways affected by azole exposure, we analyzed the expression of selected developmental genes by quantitative PCR, focusing on components of the Wnt and BMP signaling networks. As shown in Fig. 5, even low-level TRI exposure of 10 µg/L upregulated the expression of *xbra*, and *xolloid*, while *β-catenin*, *chordin* and *noggin* exhibited upward trends. These changes may reflect activation of the Wnt/β-catenin pathway and concurrent inhibition of BMP signaling via its antagonists, *chordin* and *noggin*. Upregulation of *xbra* is consistent with enhanced mesodermal induction, while *xolloid* likely represents a feedback response attempting to restore BMP activity by degrading excess *chordin*.

FLU induced a similar transcriptional profile, though the magnitude of change was generally lower and more variable. In contrast, several downstream BMP effectors—*follistatin*, *sizzled*, *vent1*, and *vent2*—did not show significant expression changes in either treatment (Suppl. Fig. 2). This selective transcriptional response indicates that azoles specifically disrupt early morphogen gradients without broadly altering all components of the BMP pathway.

### Exposure concentrations confirmed by chemical analysis

To confirm that the developmental effects observed were consistent with the intended dosing, we analyzed the actual concentrations of TRI in the exposure water using HPLC (Suppl. Fig. 3). Measurements taken during the experiment confirmed that concentrations remained stable and closely matched the nominal values. For the representative treatment groups, we selected and detected TRI at 100 μg/L, validating the accuracy and consistency of chemical exposures used in this study (Suppl. Fig. 3).

**Fig. 6:**
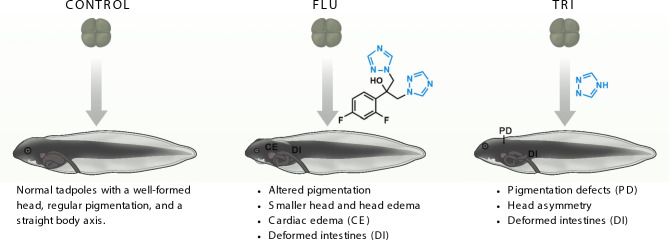
Summary of molecular and phenotypic effects of fluconazole (FLU) and 1,2,4-triazole (TRI) exposure on early *Xenopus laevis* development**.** Embryos were continuously exposed to FLU or TRI from NF stage 3 (upper row) to NF stage 45 (lower row) and subsequently analyzed, with embryos treated with vehicle solution serving as negative controls. Both azole compounds disrupt key embryonic signaling pathways, leading to upregulation (e.g., *xbra*, *xolloid)* and upward trends (e.g., *β-catenin*, *chordin*, *noggin)* in Wnt- and BMP-related genes, together with head and gut malformations, altered pigmentation, and increased heart rate. These findings highlight the developmental toxicity of azole compounds and their potential ecological impact on aquatic vertebrates. The azole ring is highlighted in light blue in the chemical structures of both compounds.

In summary, both FLU and TRI exhibit teratogenic potential in *Xenopus laevis* embryos, but through partially distinct developmental effects. FLU appears to act rapidly and reach a phenotypic plateau at low concentrations, while TRI induces malformations in a more progressive, dose-dependent manner. The spectrum of malformations and broader gene expression changes caused by TRI suggests a more pervasive disruption of axial patterning. These results, summarized in Fig. 6, collectively support the hypothesis that azole antifungals interfere with conserved signaling pathways involved in vertebrate embryogenesis, highlighting their potential risk as environmental contaminants and emerging human health hazards.

## Discussion

Our study investigates the effects of triazole-based antifungals, specifically fluconazole (FLU) and its structural core 1,2,4-triazole (TRI), on early embryogenesis in aquatic vertebrates using *Xenopus laevis* as a model. We show that these compounds selectively disrupt key developmental signaling pathways and induce distinct morphological deformities.

### Effects of FLU and its structural core TRI

Scaffold-based analyses are commonly used in medicinal chemistry to link core structures with biological activity; comparing a drug to its core scaffold is therefore an established approach for dissecting mechanism and potency differences^26^. Recent structure–activity relationship study of FLU and related analogs have shown that modifications to the TRI scaffold, for example, significantly change biological activity^27^. In mammalian models, fluconazole has also been shown to exert dose-dependent teratogenic effects. In mice, oral exposure during specific developmental windows caused cleft palate formation in up to 50% of fetuses, with a clear phase of maximal sensitivity at gestational day 10 and additional skeletal anomalies, including dysmorphic tympanic ring and shortened humerus, at higher doses^28^. Similarly, reports from chick embryo studies and other vertebrate systems further demonstrate that fluconazole exposure interferes with craniofacial and branchial development, likely through mechanisms involving disrupted neural crest migration and retinoic acid signaling^29^.

Comparable effects have also been reported in zebrafish embryos, where fluconazole exposure leads to developmental abnormalities, including craniofacial defects and reduced hatching and survival^30,31^. These findings underline the conserved nature of fluconazole-induced developmental toxicity across vertebrates and support the relevance of the *Xenopus* model used in our study. These observations align with our findings, as distinct frequency of embryotoxic effects were observed when comparing FLU with its structural core, TRI.

The most prominent embryotoxic effect we observed was an abnormal pigmentation pattern, mainly in the FLU treated condition. In embryos exposed to TRI, although this was not the most frequent effect, we noticed an interesting difference in the shape of the melanophores (data not shown). Interestingly, previous research has shown that *Xenopus* melanophores can be used as a rapid and sensitive indicator of chemical toxicity in water^32^. Another interesting malformation to note was in the mouth. Orofacial defects in *Xenopus* were already discussed by^33^, when retinoic acid signals were disrupted and median clefts that extended from the “upper lip” and into the primary palate were observed^33^, similar phenotype seen by us. Regarding the significant increase in heart rate induced by both compounds, a recent study reported that FLU causes cardiovascular toxicity in *Danio rerio*^31^. Although that study observed the opposite effect on heart rate, it employed concentrations considerably higher than those used here, which exceeds environmentally relevant exposure levels.

Taken together, the results demonstrated that FLU significantly impaired *Xenopus* embryo development, where severe malformations were observed. Although developmental defects are also seen in embryos treated with TRI, they appear with lower incidence and severity, as summarized in Fig. 3C.

### Altered gene expression

In our study, we observed the upregulation of *xbra* and *xolloid* transcripts, which provides particularly strong evidence for triazole-induced perturbations, whereas other transcripts, such as *β-catenin*, *noggin*, and *chordin*, exhibited upward trends without reaching statistical significance. Together, these results suggest a targeted rather than global reprogramming of developmental networks. The significant induction of *xbra* points to altered mesodermal specification, as it is a central regulator of mesoderm formation and posterior patterning. Its upregulation implies a shift toward posteriorized mesodermal fates, consistent with the anterior reductions observed in treated embryos^34^. The observed upregulation of *xolloid* transcripts, a metalloprotease cleaving the BMP inhibitor chordin^35^, may represent a feedback mechanism to rebalance BMP availability. Together, these transcriptional alterations suggest that TRI and FLU exposures perturbs mesodermal allocation and axial patterning through combined effects on Wnt and BMP regulatory networks. Although *β-catenin* transcript levels trended upward, it is important to note that β-catenin is regulated predominantly by protein stabilization via posttranslational modification and subsequent nuclear localization, not by transcript abundance^36^; therefore, mRNA abundance does not provide a direct readout of canonical Wnt pathway activation. Nevertheless, the observed transcriptional profile is consistent with partial Wnt pathway engagement, which could synergize with BMP modulation to reinforce posteriorization. The modest increases in noggin and chordin, two potent BMP antagonists, although not statistically significant, align with this interpretation but require cautious consideration given their variability.^37–39^.

Interestingly, *dickkopf*, *goosecoid*, *sizzled*, and *vent1/2*, were not significantly altered, suggesting that azoles exposure do not induce global transcriptional rewiring of embryonic axes, but rather target specific nodes within the Wnt–BMP regulatory interface. FLU induced similar but weaker changes than TRI, supporting a shared mechanism of action with differing potency.

### Corresponding phenotypic outcomes

These molecular alterations correlated with phenotypes including reduced head size, craniofacial defects, pigmentation abnormalities, and mild digestive tract malformations. Reduced head size likely reflects anterior tissue loss, a hallmark of posteriorizing influences on axial patterning^37^. Although β-catenin, noggin, and chordin showed only non-significant upward trends, even modest shifts in Wnt/BMP dynamics can bias tissue allocation, which may explain the anterior reductions observed in our tadpoles. Abnormal pigmentation, particularly the emergence of star-like pigment cells, is suggestive of disrupted neural crest migration^40^, which relies on tightly regulated BMP gradients^41^. Digestive tract malformations and altered heart rate are consistent with mesodermal mispatterning and cardiac lineage imbalance—both processes sensitive to Wnt/BMP signaling dynamics. Notably, similar phenotypes have been observed in zebrafish embryos exposed to other azole antifungals, including pericardial edema and craniofacial deformities^7,19^, reinforcing the idea of a conserved mode of developmental toxicity across aquatic vertebrates.

### Mechanistic links between gene expression and phenotype

Importantly, our transcriptional data provide a plausible mechanistic link between signaling disruptions and phenotype. *Xbra* is expressed in the involuting marginal zone during gastrulation and is regulated by Wnt/β-catenin signaling^42^. As a mesodermal regulator, its elevation likely shifts tissue allocation toward posterior fates. This posteriorization may be further reinforced by increased *xolloid*, which promotes chordin degradation and thus indirectly enhances BMP activity in the posterior. These molecular events help explain the anterior reductions seen in our embryos, especially in head structures and the gut.

The cardiac implications are also worth noting: although *xbra* is not directly required for cardiac gene expression, it is essential for early mesoderm formation from which cardiac mesoderm is derived^43^. Any disruptions at this stage could lead to mis-specification of cardiac tissues, potentially explaining heart malformations. Similar links between Wnt signaling and heart morphogenesis have been well established^44–46^.

### Possible involvement of oxidative stress

Previous studies have linked triazoles such as tebuconazole to oxidative stress in vertebrates^47–49^, mediated by reactive oxygen species (ROS). ROS may increase endothelial permeability^50^ and disturb osmotic balance^51^, which could explain the edematous phenotypes seen with both TRI and FLU exposure. This is consistent with previous observations of oxidative stress–related edema and cardiac abnormalities in zebrafish exposed to fluconazole^30,31^. Although ROS were not specifically tested in this study, they may represent a plausible parallel mechanism worth further investigation. Previous data also suggest that early phenotypic differences are more reliably observed in later stages of vertebrate development^52^, aligning with our decision to focus on later larval stages.

### Clinical relevance and potential signaling implications

Azole antifungals, particularly fluconazole, remain critical for treating fungal infections in immunocompromised patients, such as those with HIV/AIDS, cancer, or organ transplants^53^^,^^54^^,^^55^. *Candida albicans* remains the leading cause of systemic candidiasis, and the emergence of resistant strains like *Candida auris* poses additional challenges^56–58^. Beyond clinical use, their widespread presence in over-the-counter products such as creams and shampoos contributes to persistent environmental and human exposure.

Azoles function by inhibiting cytochrome P450 enzymes (e.g., CYP51), essential for fungal membrane synthesis^59–61^. However, these same enzymes—and the signaling cascades they modulate—are also found in vertebrate cells^62,63^. Our findings reveal that even the core structure of azoles (e.g., TRI) can perturb developmental signaling in *Xenopus* embryos, with selective effects on Wnt and BMP regulatory networks. While the precise molecular interactions remain to be elucidated, the evolutionary conservation of these pathways highlights the possibility of parallels in vertebrate development, including humans. Fluconazole and similar azole compounds may therefore represent emerging developmental and health hazards, warranting closer toxicological and clinical exploration.

## Material and methods

### Animal husbandry and embryo collection

. All procedures involving *Xenopus laevis* were conducted in accordance with Czech legislation on the use of animals for research and were approved by the relevant institutional and governmental authorities (MSMT-30784/2022 and MSMT-21426/2025, Ministry of Education, Youth and Sports of the Czech Republic; 45055/2020-MZE-18134 and 45980/2023-MZE-13143, Ministry of Agriculture of the Czech Republic; and MZP/2025/630/2482, Ministry of the Environment of the Czech Republic). The adult frogs were reared at Masaryk University, Czechia, in XenopLus (catalog number RE18001301, Tecniplast), which is an advanced, fully automated system tailored for housing amphibians, guaranteeing excellent animal care and housing conditions. The original breeding stock consisted of certified *Xenopus laevis* frogs purchased in 2020 from the European Xenopus Resource Centre (EXRC), University of Portsmouth, United Kingdom.

Embryos were obtained using standard methods. Briefly, an adult male was anesthetized in 20% MS-222 (Sigma-Aldrich, A5040) and testes were surgically removed and stored in cold 1 × Marc’s Modified Ringer’s solution (MMR; 100 mM NaCl, 2 mM KCl, 1 mM MgSO₄, 2 mM CaCl₂, 5 mM HEPES, pH 7.4) supplemented with 50 µg/mL gentamicin (Sigma-Aldrich, G3632). In all tests, we used 3 adult females and one pair of male testes. Sexually mature females were induced to ovulate via dorsal lymph sac injection of 260 IU human chorionic gonadotropin (hCG; Merck, Ovitrelle 250G), followed by overnight incubation at 18 °C. Eggs were collected the next morning by gentle squeezing and fertilized in vitro using a macerated piece of testis in 0.1 × MMR. Embryos were cultured at 18–21 °C in 0.1 × MMR and staged according to Nieuwkoop and Faber^64^.

### Assessment of lethal and sublethal endpoints (FETAX), including heart-beat measurements

Developmental toxicity was assessed following modified FETAX (Frog Embryo Teratogenesis Assay–*Xenopus*) protocols. Freshly fertilized embryos were visually inspected under a stereomicroscope (Olympus SZX7, Japan) and distributed into 24-well microplates (TPP, Switzerland), with 24 embryos per group. In test we put one embryo into one well. The embryos were exposed to three concentrations of each test compound: 1, 100, and 1000 µg/L for fluconazole (FLU), and 1, 100, and 1000 µg/L for 1,2,4-triazole (TRI). The lowest concentration was selected based on studies that measured pesticide levels in the environment, while the other two concentrations were chosen according to values used in previous research^9,10^. Each condition was tested in triplicate.

Control groups included unexposed embryos and embryos exposed to 0.1 × MMR. FLU and TRI were dissolved in 0.1 × MMR and added to the embryo culture medium, while the equivalent volume of 0.1 × MMR alone was used as a negative control. Exposure solutions including controls were renewed daily with freshly prepared media. No positive control (e.g. Cyclophosphamide) was not used in this study.

Each well contained 2 mL of the corresponding solution, which was refreshed daily to maintain > 80% of the nominal concentration. Embryos were incubated at 21 °C under a 12:12 h light/dark cycle and monitored daily until 120 h post-fertilization (hpf). Endpoints including mortality, hatching success, body length, and malformation rates were scored under a stereomicroscope. In this study, no mortality has been observed after the endpoint of the experiment, for both compounds as well as the control.

#### Heart rate measurement

Heart rate was measured in live *Xenopus laevis* tadpoles approximately 72 h post-fertilization (NF stage 40), when the heart is clearly visible through the ventral epidermis. Individual tadpoles were placed in a Petri dish containing fresh rearing medium. Cardiac activity was then observed under a stereomicroscope, and the number of visible heartbeats was counted manually for 60 s using a stopwatch. Counting was terminated after 60 s, and the recorded value was noted for each individual. A total of 12 tadpoles per condition were analyzed to calculate the mean heart rate.

### Gene expression analysis

To evaluate gene expression, two concentrations were selected for each compound (0.1 and 1 µg/L), representing the lowest exposure levels at which molecular changes could be reliably detected while minimizing developmental toxicity. The concentrations of 0.1 and 1 µg/L were used based on the environmentally relevant concentration (0.1 μg/L)^9,10^ and then a tenfold higher concentration (1 μg/L). Fertilized embryos were transferred to 6-well microplates (TPP, Switzerland), with 15 embryos per well and a total of 180 embryos per concentration.

At 120 hpf, embryos were pooled into eight biological replicates per group, with approximately 10–12 embryos (10 mg total) per replicate. Samples were transferred to 1.5 mL Eppendorf tubes, preserved in RNA later (Thermo Fisher Scientific, Czech Republic), left for 24 h at 4 °C, and stored at − 80 °C until RNA extraction.

### RNA extraction and reverse transcription into complementary DNA

Embryo samples were removed from RNA-later (Thermo Fisher Scientific, Czech Republic), dried, and homogenized with the MagNaLyser (Roche, Germany) by adding 0.5 ml of TRI Reagent RT (Molecular Research Center, USA) and 0.5 mm zirconia/silica beads (BioSpec Products, USA). After phase separation in TRI Reagent RT, total RNA was extracted and purified using the RNeasy Mini-Kit (Qiagen, Germany) following the manufacturer’s instructions. RNA concentration and purity were measured with the Nanodrop 2000 spectrophotometer (Thermo Fisher Scientific, Germany), using the 260/280 and 230/280 ratios.

The RNA was reverse transcribed into messenger RNA (mRNA) using the LunaScript RT SuperMix Kit (New England BioLabs Inc., USA), following the manufacturer’s instructions. The RNA from individual samples was diluted with RNase-free water (Qiagen, Germany) to achieve a uniform concentration of 1 μg across all samples. Control samples were prepared as recommended by the manufacturer. The 20 μl reaction mixture was first heated at 25 °C for 2 min to promote primer annealing, then at 55 °C for 10 min to synthesize complementary DNA (cDNA), and finally inactivated by heating at 95 °C for 1 min. Reverse transcription was performed using the Engine Thermal Cycler (Bio-Rad, Czech Republic). The resulting cDNA samples, including controls, were stored at − 20 °C until use in qRT-PCR.

### Primer design and quantitative real-time PCR

All samples for real-time qPCRs were conducted in triplicate using the LightCycler 480 (Roche, Germany) and the QuantiTect SYBR Green PCR Kit (Qiagen, Germany). Initially, each sample’s cDNA was diluted 1:4 with RNase-free water (Qiagen, Germany). Each 3-μl reaction contained 0.5 μl of diluted cDNA, 1 μl of primer mix, and 1.5 μl of SYBR Green Master (QuantiTect SYBR Green PCR Kit; Qiagen, Germany). The cycling protocol included an initial denaturation at 95 °C for 15 min, followed by 45 cycles of denaturation at 95 °C for 15 s, primer annealing at 58 °C for 30 s, and extension at 72 °C for 30 s. Melting analysis was performed from 60 to 95 °C. RNase-free water served as a negative control for DNA contamination and replaced cDNA templates to monitor amplification in each run. Data analysis was performed using LightCycler 480 SW 1.5 software (Roche, Germany), calculating threshold cycle (Ct) values and using the comparative Δ Ct method. The relative expression of the gene of interest (GOI) was determined with the formula: [1/(2CtGOI)]/[1/(2Ct60S)]. Normalized expression values are presented as mean ± standard deviation (SD) relative to the control.

Assays were carried out with gene-specific primers for *Xenopus laevis* (Suppl. Fig. 4). For normalization of expression data, the eukaryotic translation elongation factor 1 alpha 1 (*eef1a1*, NM_001016692.2) was used as the reference gene.

### Verification of chemical concentrations by HPLC

To confirm the actual concentrations TRI in the exposure media, water samples were randomly collected at 0 and 24 h after solution renewal and analyzed by HPLC using external standards (TRI 100 µg/L). The analysis showed that the measured concentrations closely corresponded to the nominal values (see Suppl. Fig. 3), supporting the assumption that concentrations remained stable under the same conditions across all experimental groups.

Defrosted sample of water (2 mL) was filtered through a 0.22 µm nylon filter (Millipore, USA) and used for liquid chromatography-mass spectrometry (LC/MS) analysis. A Thermo Scientific Ultra-High-Performance Liquid Chromatography (UHPLC) Accela 1250 system was connected to a Thermo Scientific TSQ Quantum Access MAX Triple Quadrupole Instrument (Thermo Scientific, USA) equipped with heated electrospray ionization probe. An Astra C_18_ (2.1 mm × 100 mm, 2.0 μm; Chromservis, CZ) column was used at a constant flow rate of 250 μL/min. For the determination of TRI, the mobile phase consisted of 0.1% water solution of formic acid (solvent A) and methanol (solvent B). The gradient used was: 0–2.0 min linear gradient from 20 to 90% B; 2.0–3.0 min held at 90% B; 3.0–4.0 min from 90 to 20% B and 4.0–5.0 min held at 20% B in order for the column to re-equilibrate before the next injection. For determination of FLU mobile phase consisted of 0.1% water solution of formic acid (solvent A) and acetonitrile (solvent B). The gradient used was: 0–3.0 min linear gradient from 40 to 90% B; 3.0–6.4 min held at 90% B; 6.4–7.3 min from 90 to 40% B and 7.3–8.0 min held at 40% B in order for the column to re-equilibrate before the next injection. The full loop injection volume of the sample was set at 2 μL. The heated electrospray ionization was operated in the positive-ion mode under the following conditions: Capillary Temperature: 325.0 °C; Vaporizer Temperature 300 °C; Sheath Gas Pressure 35.0 psi; Auxiliary (drying) gas 10 a.u.; Spray Voltage 3300 V. For our quality assurance and quality control program, the instrument was calibrated daily with multi-level calibration curves. Procedural blank and solvent blank were analysed for every set of 10 samples. The inter-day precision expressed as a relative standard deviation was 9.6% for TRI. The limit of detection determined as 3:1 signal versus noise value was 0.47 µg/L for TRI. Standards of TRI were purchased from Sigma-Aldrich (USA). Methanol and acetonitrile were purchased from Chromservis (Czech Republic) and were LC/MS purity (≥ 99.9%).

### Statistical analysis

All statistical analyses were performed using Prism 8 (GraphPad, USA). Mortality, developmental stages, and hatchability were compared using the chi-square (χ^2^) test. Continuous variables such as morphometric measurements and gene expression levels were first tested for normality (Shapiro–Wilk) and homogeneity of variance (Bartlett’s test).

If assumptions of normality and equal variance were met, one-way ANOVA followed by Dunnett’s post hoc test was applied. If assumptions were not met, non-parametric Kruskal–Wallis ANOVA followed by Dunn’s multiple comparisons test was used. Statistical significance was accepted at *, *p* < 0.05; **, *p* < 0.01; and ***, *p* < 0.001.

### Ethical statement

All experimental procedures complied with the national Act No. 246/1992 Coll. on the Protection of Animals Against Cruelty and were approved by the institutional ethics committee and relevant authorities. All procedures involving *Xenopus laevis* were conducted in accordance with Czech legislation on the use of animals for research and were approved by the relevant institutional and governmental authorities (MSMT-30784/2022 and MSMT-21426/2025, Ministry of Education, Youth and Sports of the Czech Republic; 45055/2020-MZE-18134 and 45980/2023-MZE-13143, Ministry of Agriculture of the Czech Republic; and MZP/2025/630/2482, Ministry of the Environment of the Czech Republic). The work on embryos was performed up to NF stage 45, prior to the onset of independent feeding, and therefore did not require additional ethical approval under EU Directive 2010/63 and national legislation.

## Supplementary Information

Supplementary Information.

## Data Availability

All data needed to evaluate the conclusions in the paper are present in the paper or the Supplementary Materials. Large Language Models (LLMs) were not used for the generation of scientific content in this manuscript. Occasional assistance was limited to grammar and language editing.

## Abbreviations

ANOVA: Analysis of variance
BMP: Bone morphogenetic protein
CE: Cardiac edema
DI: Deformed intestines
FETAX: Frog embryo teratogenesis assay in *Xenopus*
FLU: Fluconazole
hpf: Hours post fertilization
HPLC: High-performance liquid chromatography
MMR: Marc’s modified ringer’s solution
NF: Nieuwkoop and Faber
PD: Pigmentation defects
ROS: Reactive oxygen species
SD: Standard deviation
TRI: 1,2,4-triazole
Wnt: Wingless/integrated
