# Supplementary material for: Developmental toxicity of fluconazole and 1,2,4-triazole in Xenopus laevis
Source: Sci Rep. 2025 Dec 6;16:1443. doi: 10.1038/s41598-025-30992-5 (PMC12796290; doi:10.1038/s41598-025-30992-5)
Supplement: Supplementary file 1 — Supplementary Information. [file 41598_2025_30992_MOESM1_ESM.pdf]

SUPPLEMENTARY MATERIAL FILE TO:

Developmental toxicity of fluconazole and 1,2,4-triazole in *Xenopus laevis*

Barbora Riesova[1#], Lorena Agostini Maia[2#], Renata Hesova[1], Nikola Peskova[1,3], Petr Marsalek[1], Jana Blahova[1], Pavla Lakdawala[1], and Jakub Harnos[2✉],

[1]University of Veterinary Sciences Brno, Department of Animal Protection and Welfare & Veterinary Public Brno, Czech Republic.

[2]Masaryk University, Department of Experimental Biology, Brno, Czech Republic.

[3]Veterinary Research Institute, Department of Infectious Diseases and Preventive Medicine, Brno, Czech Republic.

[#]These authors contributed equally to this work.
[✉]Corresponding author: Jakub Harnos (harnos@sci.muni.cz), ORCID: 0000-0002-0752-9260.

This file contains 4 Supplementary Figures and 1 Supplementary Table.

Fluconazole	1 µg/L	100 µg/L	1,000 µg/L
Increased pigmentation	2	8	0
Decreased pigmentation	8	0	10
Smaller head	6	5	2
Head edema	2	2	1
Heart edema	4	3	1
Intestine malformation	6	6	2
Anterior malformation	5	5	2

1,2,4-Triazole	1 µg/L	100 µg/L	1,000 µg/L
Increased pigmentation	1	0	2
Decreased pigmentation	0	0	1
Smaller head	1	1	3
Head edema	0	0	2
Heart edema	1	0	1
Intestine malformation	1	1	3
Anterior malformation	2	2	3
Curved spine	0	1	0
Fin malformation	0	1	0

Supplementary Figure 1. Spectrum and frequency of malformations in *Xenopus laevis* tadpoles exposed to fluconazole (FLU) or 1,2,4-triazole (TRI). Tadpoles were exposed continuously from fertilization to NF stage 46 to three concentrations (1, 100, or 1,000 µg/L) of FLU or TRI. The number of individuals displaying each malformation type is shown for each concentration (n = 24 per group). Phenotype counts represent the total number of affected individuals within each treatment group. No malformations were observed in the control group.

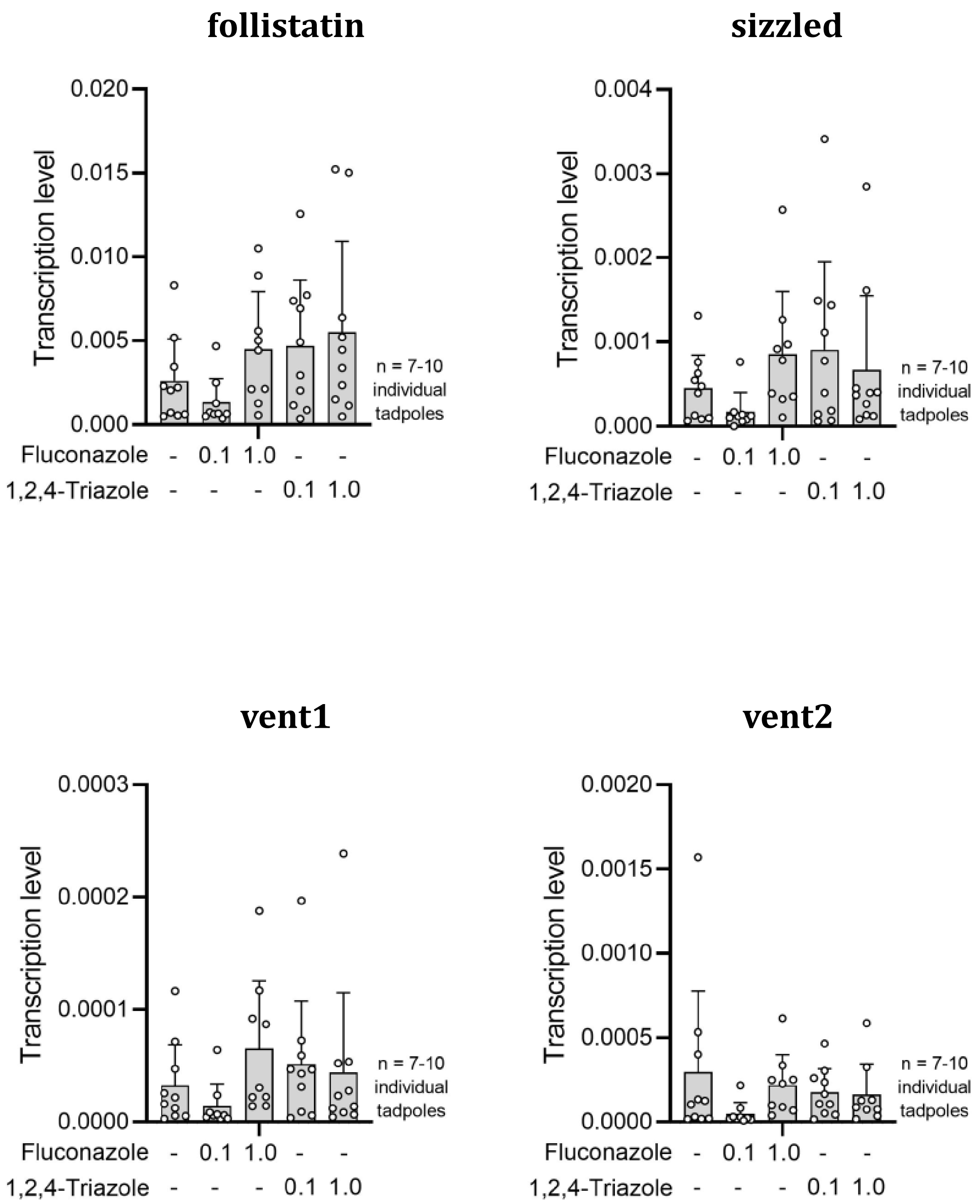

Supplementary Figure 2. Expression analysis of additional developmental genes in *Xenopus laevis* embryos exposed to FLU and TRI. No significant changes were detected in the expression of *follistatin*, *sizzled*, *vent1*, and *vent2* at 120 hpf, indicating that the observed effects are restricted to a subset of Wnt/BMP regulators. Data represent mean fold-change ± SD from 7-10 embryos. The values “0.1” and “1.0” in the graph correspond to exposure concentrations of 0.1 µg/L and 1.0 µg/L, respectively. No statistical significance using ANOVA with Dunnett’s test was found.

1,2,4–Triazole (100 µg/L)		
**Sample**	**Measured concentration (µg/L)**	
Day1_0h	104	The control samples are below the detection limit.
Day1_24h	104	
Day2_2_0h	105	**LOD (Limit of Detection):**
Day2_24h	108	**0.47 µg/L**
Day3_0h	108	
Day3_24h	104	
Day4_0h	110	
Day4_24h	112	
Day5_24h	100	

Supplementary Figure 3. Verification of chemical concentrations in exposure media. Measured concentrations of triazole (TRI) in embryo culture media closely matched nominal values, confirming stability of both compounds throughout the 5-day exposure period. Values are shown for representative doses used in FETAX experiments (100 µg/L TRI).

Primer name	Sequence (5'→3')
B-catenin fwd	AGATGCAGCAACTAAACAGGA
B-catenin rev	GTACTGCATTTTGAGCCATCT
Cerberus fwd	GCTGAACTATTTGATTTCACC
Cerberus rev	ATGGCTTGTATTCTGTGGGGCG
Chordin fwd	CCTCCAATCCAAGACTCCAGCAG
Chordin rev	GGAGGAGGAGGAGCTTTGGGACAAG
Noggin fwd	AGTTGCAGATGTGGCTCT
Noggin rev	AGTCCAAGAGTCTCAGCA
Follistatin fwd	CAGTGCAGCGCTGGAAAGAAAT
Follistatin rev	TGCGTTGCGGTAATTCACTTAC
Dickkopf-1 fwd	CACCAAGCACAGGAGGAA
Dickkopf-1 rev	TCAGGGAAGACCAGAGCA
Goosecoid fwd	CACACAAAGTCGCAGAGTCTC
Goosecoid rev	GGAGAGCAGAAGTTGGGGCCA
Xolloid fwd	GCTGGAAGTATGTGAATGGAG
Xolloid rev	GTCTTCCTGCTCCTCTGC
Xbra fwd	TTAAGTGCGGATGAGGTCC
Xbra rev	AAGTAGGGCAGAGGGGCA
Szl (Sizzled) fwd	GGCTGTGTTAGTGACCGTGA
Szl (Sizzled) rev	TCAAGCGGCCGCGATTTTTCA
Vent-1 fwd	TCCCTGCACGAGTTGCAAC
Vent-1 rev	GCATTTGGCCTGAATTTTGG
Vent-2 fwd	TGCATCTGCTCGAATTTCG
Vent-2 rev	CCTCTCTTGATGCCTGTGCCT

Supplementary Figure 4. List of primers used in this study. Sequences of primers employed for qRT-PCR and cloning experiments. Forward (fwd) and reverse (rev) primers for each gene are listed in the 5'→3' orientation.

Supplementary Table 1. Comparative physicochemical and environmental properties of fluconazole and 1,2,4-triazole.

Property	Fluconazole	1,2,4-Triazole
**Photodegradation**	Fluconazole is relatively stable to light; photodegradation occurs slowly under natural sunlight or UV exposure, indicating limited photo-reactivity.	1,2,4-Triazole is also photostable, showing only minimal degradation under UV or sunlight exposure.
**Half-life (in water/environment)**	Reported environmental half-lives range from **2 weeks to 1 year**, depending on light intensity, pH, and environmental conditions.	Half-life in aquatic environments typically ranges from **1 to 3 years**, indicating high persistence and environmental stability.
**Solubility in water**	**34.9 µg/mL** (mean of measurements at pH 7.4), indicating moderate solubility.	**1 g/100 mL** at room temperature, suggesting high solubility in water.

Supporting references to Supplementary Table 1

- Chen, Z. F., Ying, G. G., Jiang, Y. X., Yang, B., Lai, H. J., Liu, Y. S., Pan, C. H. G., & Peng, F. Q. (2014). *Photodegradation of the azole fungicide fluconazole in aqueous solution under UV-254: Kinetics, mechanistic investigations and toxicity evaluation.* **Water Research**, 52, 83–91. https://doi.org/10.1016/j.watres.2013.12.039
- Fahy, W. D., Zhang, Z., Wang, S., Li, L., & Mabury, S. A. (2025). *Environmental fate of the azole fungicide fluconazole and its persistent and mobile transformation product 1,2,4-triazole.* **Environmental Science & Technology**, 59, 3239–3251. https://doi.org/10.1021/acs.est.4c13539
- PubChem. *Fluconazole compound summary.* https://pubchem.ncbi.nlm.nih.gov/compound/Fluconazole#section=Melting-Point
- *Solubility of Things.* https://www.solubilityofthings.com/1h-124-triazole
